# Gender invariance and psychometric properties of a Brazilian version of the Emotion Regulation Questionnaire (ERQ)

**DOI:** 10.47626/2237-6089-2020-0015

**Published:** 2021-05-08

**Authors:** André Luiz de Carvalho Braule Pinto, Sonia Regina Pasian, Leandro Fernandes Malloy-Diniz

**Affiliations:** 1 Programa de Pós-Graduação em PsicologiaFaculdade de Filosofia, Ciências e Letras de Ribeirão PretoUniversidade de São PauloRibeirão PretoSPBrazil Centro de Pesquisa em Psicodiagnóstico, Programa de Pós-Graduação em Psicologia, Faculdade de Filosofia, Ciências e Letras de Ribeirão Preto, Universidade de São Paulo, Ribeirão Preto, SP, Brazil.; 2 Departamento de Psiquiatria e NeurologiaFaculdade de Medicina,Universidade Federal de Minas GeraisBelo HorizonteMGBrazil Laboratório de Pesquisa em Neuropsicologia, Programas de Pós-Graduação em Saúde da Criança e de Medicina Molecular, Departamento de Psiquiatria e Neurologia, Faculdade de Medicina, Universidade Federal de Minas Gerais, Belo Horizonte, MG, Brazil.

**Keywords:** Emotion regulation, self-regulation, psychometrics, measurement invariance, gender differences

## Abstract

**Introduction:**

Emotion regulation refers to use of strategies to change or suppress a response to an affective experience and is an important component of an individual’s subjective wellbeing. Difficulties properly regulating emotions are related to psychopathological processes.

**Objective:**

This study assessed the factor structure of the Brazilian version of the Emotion Regulation Questionnaire (ERQ); the invariance of its psychometric parameters as a function of gender; and its convergent validity with other scales measuring affective processes.

**Method:**

A total of 813 adults (73.7% women), aged between 18 and 64 years and with a high educational level used an electronic platform to complete the ERQ, the Positive and Negative Affect Schedule (PANAS), the Affect Scale (AS), and the Difficulties in Emotion Regulation Scale (DERS). Factor structure, reliability, and validity of the adapted version of the ERQ were investigated.

**Results:**

Multi-group confirmatory factor analysis (MGCFA) revealed adequate goodness of fit for the ERQ’s two-factor model (cognitive reappraisal and emotional suppression), providing evidence of invariance of its psychometric parameters as a function of gender. Correlations between the ERQ’s factors and measures of affect and emotional dysregulation presented positive indicators, with significant associations between emotion regulation and affective experiences depending on gender.

**Conclusion:**

The ERQ presents good psychometric indicators for use with the Brazilian population.

## Introduction

Emotion regulation is essential for individuals to adapt properly to changes in the environment. Emotional self-regulation can be understood as a set of cognitive and/or behavioral strategies used to control emotional experiences, whether before an emotion emerges or even when an emotion is already in course.^1^ Difficulties controlling one’s own emotions underlie negative emotional experiences and various psychological disorders. Of note, studies addressing social anxiety, personality disorder, and post-traumatic stress disorder have related such difficulties to duration, frequency, and types of emotions.^2,3^ Social risk behaviors are also related to emotion regulation^4^ and vary over the course of life according to individuals’ gender and diverse cultural contexts.^5-7^

A great variety of psychological strategies are used to balance the influence of emotion in each social context or even to modify an emotional experience entirely. These strategies are commonly grouped into two large categories: “antecedent strategies”, which focus on behaviors that precede the emergence of emotion, and “response-focused” strategies, the objective of which is to control an emotional experience already in course.^3,8^ According to Gross^3,9^ there are at least five groups of emotion regulation strategies that differ in terms of the process used to modulate affective responses or the point during the affective process at which they are implemented.

Gross & John^1^ developed the Emotion Regulation Questionnaire (ERQ) to assess individual differences related to the use of emotion regulation strategies, assessing one antecedent process (called cognitive reappraisal) and one response-focused emotion modulation process (expressive suppression). This instrument has been adapted for Germany, Italy, United Kingdom, Australia, and Spain, among a total of more than 15 countries, including Brazil.^10-14^

Studies addressing the ERQ report that, in common with positive and negative affect, which presents differences according to gender,^15,16^ different emotion regulation strategies may also be associated with a person’s gender. These differences are reported in the original study conducted by Gross & John^1^ and have been found in other cultural contexts.^5,17,18^

The findings reported so far show that socio-cultural expectations play an important role, influencing the behavioral style of emotional self-regulation among men, favoring expressive suppression strategies.^1^ The authors did not find differences between men and women regarding the use of cognitive reappraisal as an emotion regulation strategy. Later, in a large cross-cultural study involving 23 countries and 3,386 participants, Matsumoto et al.^6^ found the same emotion regulation patterns based on gender; i.e., men used emotional suppression more frequently than women. However, in a recent study with 5,000 participants from Finland, Westerlund & Santtila^18^ and also Balzarotti^19^ observed a slight difference between men and women, also in relation to the use of cognitive reappraisal strategies, with women being more likely to use that strategy. Moreover, statistically significant differences in relationships between ERQ factors were found in specific cultural groups. Cultures that are oriented toward the long term, such as Eastern cultures, tend to present a positive relationship between cognitive reappraisal and expressive suppression, while more individualistic Western cultures tend to present a negative correlation between the ERQ factors.

Therefore, the ERQ is a useful and valid instrument to support gender-based cross-cultural comparisons for emotion regulation processes. However, before conducting this type of analysis, the invariance of the existing measurement needs to be tested with respect to variables that might influence the results.

The results of an instrument such as the ERQ need to be founded on appropriate psychometric characteristics, such as invariance of psychometric parameter measures between different groups of participants or at different points in time. Measurement invariance can be defined as the probability of an individual, given his/her latent trait level, obtaining a given score, not exhibiting a change according to the group to which she/he belongs.^20^ Failure to establish measurement invariance indicates systematic bias and can be found in at least three situations: linguistic differences, when adapting an instrument to another language; instability of the theoretical model over time; and when comparing groups to assess differences in the means of latent traits.^20,21^ Only after pursuing such an investigation, can one state whether the differences found between different groups of respondents are actually due to differences in the psychological construct, without response bias given the characteristics of the instrument itself. It is therefore necessary to investigate beforehand whether an instrument’s psychometric properties do not vary depending on the individuals to whom it is administered.^21-24^

In their first study of the ERQ, Gross & John^1^ tested the instrument’s structure to see whether it remained invariant regardless of participants’ gender, which would enable comparisons between these groups, and were able to prove the measure was appropriate using confirmatory factor analysis. Later, Melka et al.^25^ also tested the invariance of the ERQ according to gender and ethnicity in a sample of 1,188 participants and confirmed the hypothesis of measurement invariance. Therefore, several studies have assessed the invariance of the instrument’s psychometric parameters, both in different cultures,^11,12,26^ and according to the participants’ gender,^25^ confirming measurement invariance.

In the Brazilian setting, the ERQ was translated by Boian et al.^13^ and afterward, Batistoni et al.^27^ presented initial evidence of its validity using exploratory factor analysis in a sample of 153 elderly individuals. The aforementioned study reported that the two-factor model explained 50.1% of data variance, with internal consistency similar to that found by the authors of the original instrument, both for cognitive reappraisal (Cronbach’s alpha = 0.74) and expressive suppression (Cronbach’s alpha = 0.69). Additionally, as expected, a positive and significant correlation was found with positive and negative affect and also with satisfaction with life.

Still in Brazil, Gouveia et al.^28^ sought to assess indicators of the ERQ’s internal validity in two studies with students. An exploratory factor analysis performed in the first study (n = 212) revealed a three-factor structure according to Kaiser and Horn’s criteria (parallel analysis) and excluded item 9. The internal consistency of these three factors was lower than expected, with Cronbach’s alphas ranging from 0.60 to 0.66. A confirmatory factor analysis was performed in the second study (n = 229), to compare the model found with a one-factor model and the two-factor model. Although the goodness of fit indexes for the three-factor model were superior to the others, the AGFI and CFI indexes fell short of recommendations in the international literature.^29,30^ The authors therefore suggested that future studies should assess the adequacy of the theoretical model proposed for the ERQ in Brazil.

This study was devised to address the disparate results reported by Brazilian studies regarding the ERQ’s factor structure and the lack of studies testing the gender invariance of its psychometric parameters. The objective is to contribute to research addressing the factor structure of the Brazilian version of the ERQ, by assessing the invariance of its parameters according to gender; investigating how accurate the measure is by checking its internal consistency and temporal stability; and finding evidence of the ERQ’s validity based on measures of affect and emotional dysregulation.

The Brazilian version of the ERQ is expected to present indicators of invariance with respect to gender, with good accuracy indexes for both its factors. Additionally, the cognitive reappraisal factor is expected to be associated with positive affect and inversely related to negative affect. These hypotheses derive from the findings reported by Batistoni et al.^27^ and John & Eng,^10^ which suggest cognitive reappraisal influences an individual’s affective experience, being linked to subjective wellbeing and satisfaction with life.

Likewise, expressive suppression is expected to be significantly related to negative affect and inversely related to positive affect. Considering that emotional suppression is a late strategy of emotion regulation, that is, it is implemented when the affect is already present, individuals using it are expected to experience negative affect. Empirical evidence shows that this strategy (expressive suppression) is ineffective at modifying an existing emotion.^1,31^

An additional hypothesis that is tested in this study concerns a potential poor correlation between use of emotion regulation strategies and presence of affect dysregulation, according to information provided by the Difficulties in Emotion Regulation Scale (DERS).^32^ According to John & Eng,^10^ even though these are apparently similar phenomena, or even opposed phenomena,^6^ emotion dysregulation (assessed using the DERS) is related to diversified processes, which are not always directly linked to cognitive reappraisal or expressive suppression strategies and may thus be considered different phenomena. Recent studies have shown a poor correlation between ERQ and DERS indicators, which provides evidence that they deal with different processes.^18,33^ This hypothesis, if confirmed, would contribute to evidence of the ERQ’s discriminant validity in Brazilian samples.

## Materials and method

This is an empirical study with a cross-sectional, descriptive, and inter-group comparative design. Ethical guidelines were considered in all stages of the study and the institutional review board approved the research project (CAAE 62744516.3.0000.5407).

### Participants

A total of 813 adult volunteers agreed to participate in the study online. They were aged between 18 and 64 years (mean = 27.5; standard deviation [SD] = 9.1); had a high educational level (mean = 15.1 years of schooling; SD = 4.7; Min = 1; Max = 21); and 73.6% were women (n = 609), while 26.4% were men (n = 214). The most frequent marital status category was single (70.3%), followed by married or in a stable relationship (22.7%), divorced (3.0%), and widowed (0.5%). Most participants belonged to a middle-income class (levels B or C), according to the Brazil Economic Classification Criterion,^34^ which also conforms to their educational level. Thus, the study sample is characterized by young adults with a high educational level and a predominance of women.

### Instruments

The participants individually completed the following psychological assessment instruments online:

- Emotion Regulation Questionnaire (ERQ): developed by Gross & John^1^ and translated into Brazilian Portuguese by Boian et al.^13^ This is a 10-item instrument scored on a Likert scale ranging from 1 (total disagreement) to 7 (total agreement) and was designed to assess two emotion regulation strategies: expressive suppression and cognitive reappraisal. Batistoni et al.^27^ assessed elderly Brazilian individuals and reported good accuracy indicators, verifying internal consistency with Cronbach’s alpha (Cognitive reappraisal = 0.73; Expressive suppression = 0.69) and test-retest coefficients (Cognitive reappraisal: r = 0.75; Expressive suppression r = 0.73).
- Positive and Negative Affect Schedule (PANAS): developed by Watson et al.^35^ This scale was adapted for Brazil by Giacomoni & Hutz^36^ and later refined and standardized by Zanon & Hutz.^37^ The PANAS is a self-report instrument on which respondents are asked to report how intensely they feel emotions described by 20 adjectives on a Likert scale ranging from 1 (not at all) to 5 (extremely). It has Cronbach’s alphas of 0.86 for positive affect and 0.88 for negative affect.
- Affect Scale (AS): developed in Brazil by Zanon et al.^38^ The AS is composed of 20 statements describing past and current feelings and emotions, assessing two factors: positive affect and negative affect. The scale presented good convergent and divergent validity, as well as good internal consistency, with Cronbach’s alphas of 0.83 for positive affect and 0.77 for negative affect.
- Difficulties in Emotion Regulation Scale (DERS): developed by Gratz & Roemer.^39^ This scale comprises 36 statements addressing emotions that are rated on a 5-point Likert frequency scale. The DERS assesses six dimensions of difficulties regulating emotions, namely: a) lack of access to strategies used to control emotions (Strategies); b) lack of clarity about emotions (Clarity); c) poor knowledge of emotions (Awareness); d) difficulty controlling impulses (Impulse); e) difficulty engaging with objectives (Objectives); and f) not accepting emotional responses (Non-acceptance). In Brazil, Miguel et al.^32^ investigated the DERS’ psychometric properties and found good indicators of accuracy and of validity, with Cronbach’s alphas ranging from 0.77 to 0.93.

### Procedures

After the institutional review board had approved the project, the study was released on social media and its objectives and tasks were disclosed. Those interested in participating received a link via which they accessed free and informed consent forms, confirmed their agreement, and completed the instruments along with a sociodemographic questionnaire. Participants were also informed that they would complete a retest 15 days later. The platform recorded 1,235 accesses and those who did not fully complete the instruments were excluded, so that a total of 813 participants remained. After 15 days, the participants received an electronic message with a new link to complete the retest and 225 participants completed this second stage.

Data were analyzed using R (version 3.5) available free of charge at https://cran.r-project.org/, together with the lavaan,^40^ semTools^41^ and psych^42^ packages. Data were first systematized using descriptive statistics for both sociodemographic variables and those concerning the psychological instruments following their respective technical standards.

Next, indicators of the validity of the ERQ were verified with confirmatory factor analysis (CFA), using the robust weighted least squares (WLSMV) estimator, root mean square error of approximation (RMSEA), comparative fit index (CFI), Tucker-Lewis index (TLI), and standardized root mean square residual (SRMR), using the cut-off points suggested by Hu & Bentler^30^ and DiStefano^29^ as the criteria for model fit. After confirming the ERQ factor model, a multi-group confirmatory factor analysis (MGCFA) was performed to verify the invariance of the ERQ parameters in respect to the participants’ gender. We compared latent mean differences between gender using a full residual invariance model as baseline. To compare latent mean between genders, we constrained the men’s group latent mean to 0 and leaving latent means for the women’s group free to estimate. We used the value of the critical ratio to assess latent mean differences, whereby a value larger than 1.96 indicates statistically significant differences in latent means. After that, we calculated an effect size based on Hancock’s suggestions.^43^

Afterwards, reliability was estimated in three different ways: Cronbach’s alpha was used to verify internal consistency and test-retest temporal stability was assessed using Pearson’s correlation coefficient and the intraclass correlation coefficient (ICC). Finally, we checked potential correlations with the PANAS, AS, and DERS variables; i.e., we tested for associations between the ERQ factors and positive and negative affect (PANAS and AS) and emotion dysregulation (DERS).

## Results

**Figure 1:**
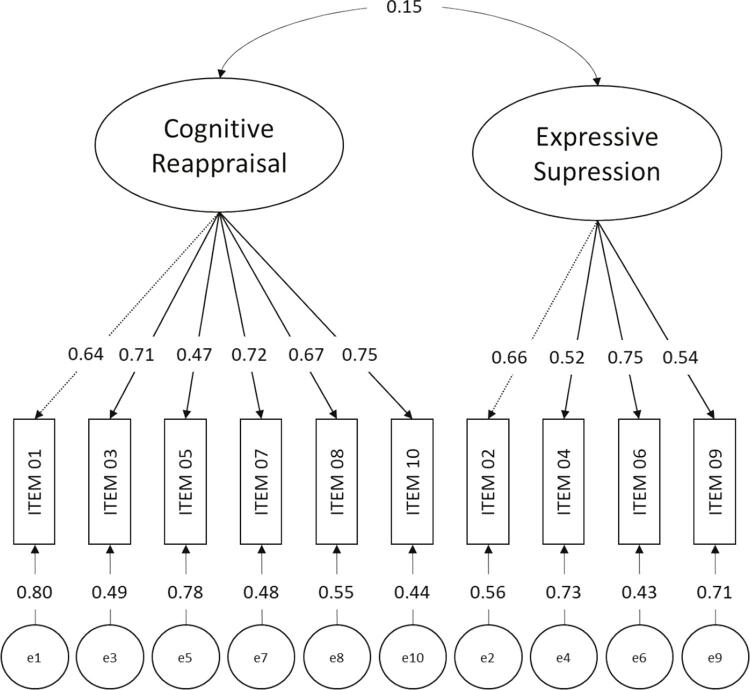
Factor analysis of the Emotion Regulation Questionnaire (ERQ)

The two independent factor model proposed by the authors of the original ERQ was tested first. The results of the confirmatory factor analysis (CFA) are illustrated in Figure 1.

The results show that the ERQ’s two-factor model has adequate goodness of fit indexes (χ^2^[34] = 74.190; GFI = 0.991; CFI = 0.982; TLI = 0.977; RMSEA = 0.038; 90% confidence interval [90%CI] = 0.026-0.050; SRMR = 0.049), which allowed us to proceed with the MGCFA in order to answer the question of whether the ERQ Brazilian version is invariant in respect to the respondents’ gender. The results of this second analysis are presented in Table 1.

**Table 1:** Multi-group confirmatory factor analysis: total model and invariance tests by gender

Model	χ^2^(df)	Δχ^2^(df)	SRMR	TLI	CFI	ΔCFI	RMSEA	90%CI	ΔRMSEA
Women	62.634 (34)	-	0.052	0.975	0.981	-	0.038	0.022-0.052	-
Men	22.445 (34)	-	0.050	0.999	0.999	-	0.000	0.000-0.013	-
Configural invariance	85.080 (68)	-	0.047	0.990	0.993	-	0.025	0.000-0.040	-
Metric invariance	95.788 (76)	10.708 (8)	0.050	0.990	0.991	0.002	0.025	0.000-0.040	0.000
Structural invariance	108.308 (84)	12.52 (10)*	0.052	0.989	0.989	0.002	0.027	0.007-0.040	0.002
Residual invariance	126.174 (94)	17.866 (2)*	0.057	0.987	0.986	0.002	0.013	0.013-0.041	0.014
Mean variance	184.742 (98)	58.568 (4)	0.067	0.965	0.962	0.024	0.047	0.036-0.057	0.034

90%CI = 90% confidence interval; CFI = comparative fit index; df = degrees of freedom; RMSEA = root mean square error of approximation; SRMR = standardized root mean square residual; TLI = Tucker-Lewis index.

* p < 0.01.

Note that all the models tested (configural, metric, structural, and residual) adequate presented goodness of fit indexes when we considered ΔCFI and ΔRMSEA, but not when the chi-square difference (Δχ^2^) was considered. According to Damásio,^22^ and Cheung & Rensvold,^44^ this index may change according to sample size, tending to be sensitive to this characteristic, while the ΔCFI might be a more appropriate indicator, because it cannot exceed 0.015. Thus, these results support the invariance of ERQ parameters in respect to the gender of the participants.

Measurement reliability for both the ERQ factors was assessed using Cronbach’s alpha coefficients and test-retest correlation coefficients (Pearson’s r and ICC). The Cronbach’s alpha for cognitive reappraisal factor was 0.83 and test-retest results were r = 0.66 for Pearson’s correlation and ICC = 0.80, while the alpha for expressive suppression was 0.71 and test-retest results were r = 0.75 for Pearson’s correlation coefficient and ICC = 0.86. Thus, we can empirically attest to the internal consistency of both ERQ factors and the temporal stability of ERQ results, noting that the Brazilian version of the ERQ presents adequate indicators.

Next, the relationships between ERQ factors and affect variables, related to positive and negative affect, and also between ERQ factors and emotion dysregulation variables, were assessed. These findings are presented in Table 2.

**Table 2:** Analysis of correlation between ERQ, PANAS, AS, and DERS

Instrument/variable	ERQ	
	Cognitive reappraisal	Expressive suppression
PANAS		
Positive affect	0.342*	-0.187*
Negative affect	-0.284*	0.114*
AS		
Positive affect	0.407*	-0.121*
Negative affect	-0.324*	0.132*
DERS		
Awareness	0.205*	-0.082^†^
Clarity	-0.084^†^	0.158*
Objectives	-0.189*	-0.012
Impulse	-0.224*	0.005
Strategies	-0.269*	0.089^†^
Non-acceptance	-0.137*	0.101*

AS = Affect Scale; DERS = Difficulties in Emotion Regulation Scale; ERQ = Emotion Regulation Questionnaire; PANAS Positive and Negative Affect Schedule.

* Significant correlation p < 0.01 (two-tailed).

^†^ Significant correlation p < 0.05 (two-tailed).

The results indicate positive and statistically significant associations between the ERQ’s cognitive reappraisal and positive affect assessed by the PANAS and AS. An inverse relationship was also found between negative affect and the cognitive reappraisal strategy (PANAS and AS). As expected, the ERQ’s expressive suppression factor was statistically correlated with both positive and negative affect, though with a lower level of correlation.

As hypothesized, the results revealed negative and statistically significant correlations between all emotion dysregulation (DERS) factors and ERQ cognitive reappraisal, with the exception of the awareness factor. Correlations with the ERQ expressive suppression strategy were close to zero, indicating a weak association between suppression of emotions and emotion dysregulation (as assessed by the DERS). Together, these results indicate a convergence of the processes assessed by the ERQ and the affective traits assessed by the PANAS and AS, but with a poor relevant association with emotion dysregulation.

Finally, we sought to characterize emotion regulation indicators (ERQ) in this study sample, testing the hypothesis of potential differences in latent means as a function of the participants’ gender. Based on the full residual invariance across gender, we can compare the latent mean differences. The results indicated that women had a slightly higher latent mean than men for Cognitive Reappraisal (critical ratio = 0.093; p < 0.001; effect size = 0.09), and a lower mean for Emotional Suppression (critical ratio = -0.343; p < 0.001; effect size = 0.34). As expected, differences were found in use of the emotion suppression strategy; men used this strategy more frequently than women. Although a statistical difference in cognitive reappraisal was observed, the effect size was negligible for practical purposes. No statistically significant differences were found in any of the remaining study variables based on comparisons between genders.

## Discussion

The main objective of this study was to investigate the psychometric properties and invariance of the ERQ parameters in a Brazilian sample, testing the potential influence of participants’ gender. We also investigated evidence of validity and accuracy estimates of the ERQ at a national level.

Evidence has been presented in the scientific literature of a different composition of items in the two-factor theoretical structure originally proposed for the ERQ.^11^ However, there are also other studies suggesting that the ERQ was invariant in regard to samples of different nationalities,^12^ but not among groups with important clinical symptoms.^4^ It was important to verify the composition of the ERQ when tested with a sample of Brazilian individuals. The findings reported here confirm the two-factor structure, with adequate goodness of fit according to criteria proposed in the international literature.^29,30^ These results are in agreement with findings reported by Batistoni et al.^27^ from a sample of elderly Brazilian individuals. However, they diverge from those reported by Gouveia et al.,^28^ who assessed a sample of students using exploratory analysis and found three factors instead of the two factors predicted by the authors of the original ERQ. This divergence may have been because of the methods used to establish the number of factors.

Skinner et al.^45^ conducted a review of the scientific literature to resolve divergences concerning the factor structure of coping strategies and showed that exploratory methods tend to present divergent factor solutions, depending on the samples analyzed. This has been confirmed by simulation studies conducted to estimate the number of factors of various other psychological instruments.^46,47^ Keith et al.^47^ recommend using confirmatory factor analysis (CFA) because it is a theory-based analysis that is more restrictive and therefore has greater potential to clarify divergences in terms of the latent structures of assessment instruments. Therefore, based on both absolute (SRMR and RMSEA) and comparative (CFI and TLI) fit indexes and considering the use of a numerically relevant sample, we infer that the results support the original model of two ERQ factors.

MGCFA showed that the psychometric properties of the ERQ exhibit invariance at the configural, metric, structural and residual levels when the ΔCFI difference is taken into account, but not when differences in the chi-square test (Δχ^2^) are considered. According to Damásio,^22^ and Cheung & Rensvold,^44^ χ^2^ tends to be influenced by small differences in the matrix of covariance between groups, as well as by sample size. Given that the female and male groups had different sample sizes in this study and considering that CFI has been identified as one of the most promising indexes for identifying invariance of parameters,^44^ we can state that the psychometric properties of the ERQ are invariant for the sample assessed here.

After confirming the invariance of the ERQ in respect to gender, we verified its internal consistency using Cronbach’s alpha coefficients, which are indicative of accuracy and validity.^48^ According to the literature, alphas above 0.7 are considered adequate for psychometric measures.^49,50^ Thus, our findings confirm the accuracy of the Brazilian version of the ERQ, with alphas of 0.83 for cognitive reappraisal and 0.71 for expressive suppression. Additionally, test-retest correlations were within the acceptable range, 0.66 and 0.73 for reappraisal and suppression, respectively, and the ICC between scores at the two ERQ administration times also confirmed the stability of the scores across time. These results are similar to those reported by other psychometric studies of the ERQ, both from international studies^11,14^ and from a Brazilian study conducted by Batistoni et al.,^27^ which contradicted the three-factor model obtained by Gouveia et al.^28^ Therefore, the two-factor ERQ model is more consistent than the three-factor model, offering greater measurement reliability.

After confirming the ERQ’s factor structure and reliability, the latent mean scores obtained by men and women were compared to verify potential differences in their use of emotion regulation strategies. The results indicated that, in general, men suppress their emotions more frequently than women. Regarding the use of cognitive reappraisal, it was observed that women tend to have higher scores than men. However, similar results were reported by Westerlund & Santtila^18^ in a wide sample of Finnish participants, but the difference observed was small, and also by Balzarotti^19^ in an Italian sample. These results are in agreement with those reported by other studies using the ERQ in different cultures.^1,5^ Neuroimaging studies have shown that these differences take place at a functional level, hypothesizing that the brains of men and women process information of an emotional nature differently.^7,51,52^ It is not clear, however, what role culture plays in how these differences are established, considering that differences related to participants’ gender are even found in Asian groups, among whom more frequent use of emotion suppression is expected.^5^

As predicted, a statistically significant positive association was found between cognitive reappraisal strategy and positive affect, as assessed by the PANAS and the AS. An inverse relationship was also found with negative affect on both scales. These results agree with the international and Brazilian literature,^27^ which relates use of emotion regulation strategies to greater subjective wellbeing and reduced presence of negative affect. These findings may have important implications for interventions intended to improve the subjective wellbeing of individuals undergoing psychological treatment, considering that interventions based on modification of emotion regulation strategies tend to elicit positive results in the long term.

A positive and statistically significant correlation was found between use of expressive suppression and negative affect, as well as an inverse correlation with positive affect. These relationships, however, are close to zero, which can be explained by the fact that individuals use expressive suppression relatively late in emotional processing.^8,31^ Presence of expressive suppression thus seems to have little impact on an already ongoing affective experience, only changing expression of emotion, rather than the feeling experienced. It is often considered a poor strategy from the perspective of efficiency, in addition to negatively impacting emotional balance in the long run.

Most of the correlations found between the ERQ and the DERS were close to zero, while cognitive reappraisal was negatively related to difficulties in emotion regulation, with the exception of the awareness factor, which was positively correlated (r = 0.205). Bardeen et al.^53^ assessed the psychometric properties of the DERS in 1,045 American undergraduates and reported that the awareness factor seemed to diverge from the remaining factors, so there is a chance it is not adequate for the model proposed by the authors of the original scale. Hence, the relationship found here may reflect such divergences already reported in literature addressing the DERS.

Again, the expressive suppression factor was poorly related to the same difficulties in emotion regulation. In a recent study, Zelkowitz & Cole^33^ identified a pattern of correlations between ERQ and DERS indicators with values between 0.07 (emotional suppression and impulses) and 0.34 (emotional suppression and awareness). In another study, Westerlund & Santtila^18^ observed a similar pattern, but with weaker correlations between the two scales. These findings suggest the phenomena assessed by these scales (ERQ and DERS) are independent. Thus, we conclude that the ERQ and the DERS assess opposed phenomena, considering that the DERS is commonly used by international studies as a measure of emotion regulation. John & Eng^10^ argue that while the DERS focuses on skills (more precisely, the absence of skills) to assess an individual’s competence in regulating emotions, the ERQ focuses on two strategies that can be used on a daily basis (reappraisal and expressive suppression), not linked to any specific context. In short, the results concerning the ERQ and DERS present evidence of validity.

## Final considerations

The objective of this study was to assess the psychometric qualities of the ERQ and its invariance in respect to the respondents’ gender for a Brazilian sample. The results show that the two-factor structure proposed by the authors of the original ERQ is maintained in the Brazilian version of the scale, both according to the factor model’s goodness of fit indexes and according to its adequate relationship with other affective measures and also according to indicators of accuracy based on internal consistency and temporal stability. As expected, differences were found regarding use of the expressive suppression strategy on the part of men, corroborating the results reported in international studies.

Even though the data presented contribute to analysis of emotion regulation among adults, providing evidence of the validity and accuracy of the ERQ for use in Brazilian settings, some limitations should be noted. This study was conducted on an online platform using a convenience sample and so the conditions under which the participants completed the questionnaires could not be controlled. Therefore, this study needs to be replicated with face-to-face samples to verify the potential influence of the method by which data are collected. Additionally, further studies addressing different groups of individuals are needed, because the evidence found so far indicates a possibility that the ERQ is not appropriate for use among adolescents and children or with clinical samples.
